# Attapulgite–MXene Hybrids with Ti_3_C_2_T_x_ Lamellae Surface Modified by Attapulgite as a Mechanical Reinforcement for Epoxy Composites

**DOI:** 10.3390/polym13111820

**Published:** 2021-05-31

**Authors:** Lu Liu, Guobing Ying, Yinlong Zhao, Yuexia Li, Yiran Wu, Dong Wen, Meng Wu, Minghui Wang, Qingzhong Zhou, Xiang Wang, Cheng Wang

**Affiliations:** 1 Department of Materials Science and Engineering, College of Mechanics and Materials, Hohai University, Nanjing 211100, China; liulu201709@163.com (L.L.); loong1016@126.com (Y.Z.); liyuexia0428@163.com (Y.L.); ran459965282@163.com (Y.W.); wumeng32323@163.com (M.W.); wangmh201809@163.com (M.W.); zhouqingzhong109@163.com (Q.Z.); wangch@hhu.edu.cn (C.W.); 2 Key Laboratory of Superlight Materials & Surface Technology, Ministry of Education, Harbin Engineering University, Harbin 150001, China; wendonghhu@163.com (D.W.); wangxiang@hrbeu.edu.cn (X.W.)

**Keywords:** MXene Ti_3_C_2_T_x_, attapulgite, hybrids, epoxy-matrix composites, mechanical properties

## Abstract

As a member of two-dimensional (2D) materials, MXene is an ideal reinforcement phase for modified polymers due to its large number of polar functional groups on the surface. However, it is still relatively difficult to modify any functional groups on the surface of MXene at present, which limits its application in enhancing some polymers. Herein, one-dimensional (1D) attapulgite (ATP) nanomaterials were introduced onto the surface of MXene to form ATP–MXene hybrids, which successfully improved the mechanical properties of the epoxy composites. ATP with appropriate content can increase the surface roughness of the MXene lamellae to obtain better interface interaction. Therefore, remarkable enhancement on the mechanical property was achieved by adding M02A025 (0.2 wt % MXene and 0.25 wt % ATP), which is the optimum composition in the hybrids for composite mechanical properties. Compared to neat epoxy, the tensile strength, flexural strength and critical stress intensity factor (*K_IC_*) of M02A025/epoxy are increased by 88%, 57%, and 195%, respectively, showing a high application prospect.

## 1. Introduction

Nanoscale 2D materials are ideal reinforcement phases for strengthening and toughening polymer materials due to the high specific surface area and excellent mechanical properties [1,2,3,4,5]. As a fast-developing branch of 2D materials, MXene is a transition metal carbide and/or nitride with a graphene-like structure [6]. Due to the unique 2D layered structure, large specific surface area, excellent mechanical properties, rich surface functional groups, and light transmittance [7,8,9,10], MXene has been widely used in the field of reinforced polymer composites [11,12,13,14,15,16,17]. The general formula of MXene is M_n+1_X_n_T_x_ (n = 1, 2, 3 or 4), M refers to an early transition metal, and X refers to C or N elements [18,19,20,21]. The T_x_ in the formula represents the surface terminations, such as O, OH, F, and/or Cl, bonded to the outer M layers [22,23,24,25]. Surface modification is a way to expand reinforcing phases, increase their compatibilities with specific polymers and enhance the reinforcing effect [26]. However, the current preparation of MXene is mainly to immerse the MAX phase [27] in hydrofluoric (HF) acid or hydrochloric (HCl) acid solution dissolved LiF to etch away the A-layer elements selectively [28], which leads to the exposed M layer during the etching process directly combine with free radical groups (OH, O, F, etc.) in the solution. Moreover, due to MXene is easily oxidized in an aqueous solution [29,30], MXene is relatively difficult to change the composition of terminations [25], which undoubtedly severely limits the wider application of MXene in the field of reinforced polymers.

In addition to changing the surface functional groups of the reinforcement, another promising strategy is to use a hybrid filler composed of two or more filler materials [31,32,33], such as grafting a 1D material onto the surface of a 2D material to obtain a hybrid filler. From a practical point of view, the more accessible 1D materials and the lower the cost, the easier it is to realize applying the hybrid-reinforced polymer. Attapulgite (ATP) is a kind of typical 1D material whose chemical formula Si_8_O_20_Al_2_Mg_2_(OH)_2_(OH_2_)_4_(4H_2_O) [34]. As a natural 1D nano mineral material, ATP has many advantages, such as large specific surface area, good mechanical strength, high thermal stability and low cost, and is used in catalysis, adsorption and other fields [35,36]. According to the report [26], there are many OH functional groups on the surface of ATP, making it easy to connect with the surface of 2D materials through hydrogen bonds to form hybrids.

The main purpose of this paper is to combine 1D ATP nanorods and 2D MXene nanosheets to form ATP–MXene hybrids and use them to form a novel hybrid-reinforced epoxy based on our previous research on Ti_3_C_2_T_x_ (MXene) reinforced epoxy [37]. This combination can further improve the mechanical properties of epoxy matrix composites and broaden the applications of MXene in the field of reinforced polymers. In this work, the amount of MXene added was fixed at 0.2 wt % (In our previous work [37], in the epoxy system of diglycidyl ether of bisphenol A (DGEBA)/methyl tetrahydrophthalic anhydride (MTHPA), 0.2 wt % MXene/epoxy composite has the best mechanical properties). On this basis, composites with different contents of ATP were prepared. The results show that ATP added with an appropriate amount can uniformly be adsorbed onto the surface of the MXene layer. The hybrids further improve the mechanical properties of the epoxy, showing a good application prospect. The composites’ thermal properties and mechanical properties were comparatively studied, and the influence of different ATP contents on the mechanical properties of the composites was discussed.

## 2. Experimental

### 2.1. Materials

Commercial Ti powder (99.5 wt %, −45 μm, Jinzhou Institute of Metal Material, Jinzhou, China), Al powder (99.7 wt %, −29 μm, China Northeast Light Alloy Co., Harbin, China), and TiC powder (99 wt %, 2~4 μm, Aladdin Industrial Co., Shanghai, China) were used as raw materials. Lithium fluoride (LiF, 99 wt % purity), hydrochloric acid (HCl, AR), acetone, and anhydrous ethanol were all provided by Sinopharm Chemical Reagent Limited Corporation (Shanghai, China). DBEGA was purchased from Baling Company, SINOPEC (Yueyang, China). MTHPA and 2,4,6-Tris (dimethylaminomethyl) phenol (DMP-30) were from Tianjin Chemical Co. (Tianjin, China). Deionized water with a resistivity of >18 MΩ·cm was prepared by the TS-RO-10 L/H ultrapure water system (TAOSHI water equipment Engineering Co., Ltd. (Dongguan, China). ATP (>98 wt % purity) was from Dingbang Co., Ltd. (Changzhou, China).

### 2.2. Synthesis of Composites

**Figure 1:**
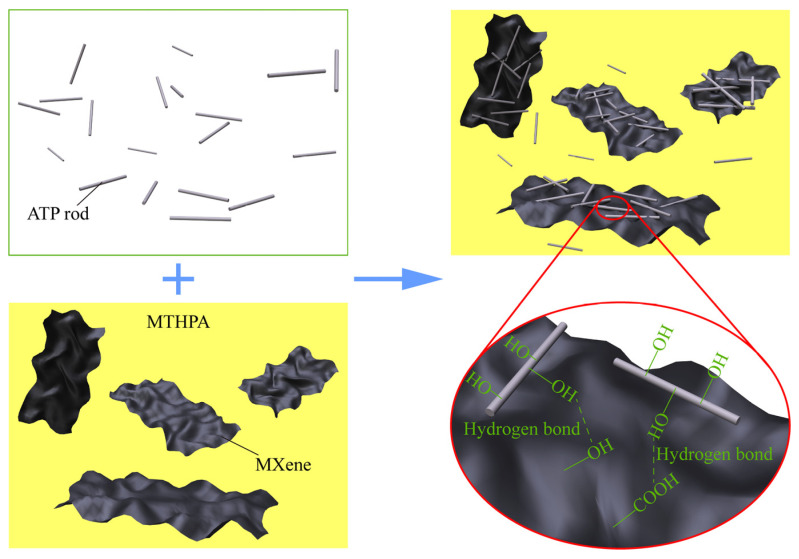
Schematic image of the dispersion and combination of hybrids in an epoxy matrix.

Comprehensive details regarding the preparation of Ti_3_AlC_2_ and Ti_3_C_2_T_x_ are provided in our earlier papers [37,38,39]. The MTHPA dispersion of Ti_3_C_2_T_x_ was prepared according to the mass fraction of MXene is the composite of 0.2%. Then, ATP was added to the MTHPA dispersion of Ti_3_C_2_T_x_ and ultrasonicated for 2 h. During the ultrasonicated treatment process, ATP can be connected to the surface of MXene through hydrogen bonds [26], as shown in Figure 1. Finally, DGEBA was added according to the mass ratio of MTHPA to DGEBA at 85:100, and the mixture was poured into the mold to solidify. The curing conditions were 1 h at 90 °C and 4 h at 110 °C differently. To reduce the curing time, 0.3 wt % DMP-30 was added. The combinations for ATP–MXene hybrids were 0.1 wt % ATP + 0.2 wt % MXene, 0.25 wt % ATP + 0.2 wt % MXene, 0.5 wt % ATP + 0.2 wt % MXene and 1 wt % ATP + 0.2 wt % MXene, respectively, based on the weight fraction of ATP in composites. Accordingly, the composites were denoted as M02A01/epoxy, M02A025/epoxy, M02A05/epoxy and M02A10/epoxy, respectively, representing the composition of hybrid filler and the matrix material. The applied formulations are shown in Table 1. The data of pure epoxy samples and 0.2 wt % MXene/epoxy (M02/epoxy) composites reported in earlier work [37] were compared.

### 2.3. Characterization

Thermogravimetric analyses (TGA, NETZSCH STA 449 F3, Selb, GER) of neat epoxy and composites were carried out under a nitrogen atmosphere from 25 to 790 °C at a heating rate of 10 °C min^−1^. The storage modulus and loss factor (tanδ) as a function of temperature were determined via single cantilever mode of the dynamic mechanical analyzer (DMA, TA Q800, New Castle, DE, USA) in the temperature range from 25 to 200 °C at 3 °C min^−1^ under air atmosphere, frequency of 1 Hz, and maximum amplitude of 0.1%. The specimen dimension was kept at 30.0 mm long × 10.0 mm wide × 5 mm thick. The glass transition temperature was obtained by using the maximum tanδ. The crosslinking density ($V_{e}$) was calculated by Equation (1) [40] as follows:

(1) $$V_{e}=\frac{E_{r}}{3\varphi RT}$$

where $E_{r}$ is the storage modulus, R is the gas constant, T is the absolute temperature, and φ is the front factor. The storage modulus at T_g_ + 50 °C was used to calculate the crosslinking density [41]. The φ varying in the range of 0.4–1.6, therefore, a value of φ = 1 was used for a good order-of-magnitude prediction [42].

The tensile and flexural tests were performed at room temperature using an Instron 3367 mechanical testing machine (Instron Co., Ltd., Canton, MA, USA) following ISO 527-1:1993 and ISO-178-2010, respectively. Standard dumbbell-shaped specimens (75.0 mm long × 12.5 mm wide × 2.0 mm thick) with a length of the narrow region of 25 mm were prepared for tensile testing at a rate of 1.0 mm min^−1^. Rectangular specimens (60.0 mm long × 8.0 mm wide × 3.0 mm thick) were used for flexural testing and loaded with a span of 48 mm at a crosshead speed of 2.0 mm min^−1^. At least five specimens were tested under each set of conditions. The tensile strength (σ) was calculated by Equation (2) [43]:

(2) σ=FA

where *F* is the measured force concerned, *A* is the initial cross-sectional area of the specimen. The elastic modulus (*E*) was calculated by Equation (3) [43]:

(3) $$E=\frac{\sigma_{0.25\%}-\sigma_{0.05\%}}{\varepsilon_{0.25\%}-\varepsilon_{0.05\%}}$$

where $\sigma_{0.25\%}$ and $\varepsilon_{0.25\%}$ are the measured tensile strength and strain at the strain value ε=0.25%, $\sigma_{0.05\%}$ and $\varepsilon_{0.05\%}$ are the measured tensile strength and strain at the strain value ε=0.05%. The flexural strength ($\sigma_{fM}$) and flexural strain ($\varepsilon_{fM}$) was calculated by Equation (4) [44] and Equation (5) [44] as follows:

(4) $$\sigma_{fM}=\frac{3FL}{2bh^{2}}$$

(5) $$\varepsilon_{fM}=\frac{6sh}{L^{2}}$$

where *F* is the maximum load of the load–displacement curve of the bending specimens, *L* is the span, *b* is the width of the sample, *s* is the deflection, *h* is the thickness of the sample. The flexural modulus ($E_{fM}$) was calculated by Equation (6) [44]:

(6) $$E=\frac{\sigma_{fM0.25\%}-\sigma_{fM0.05\%}}{\varepsilon_{fM0.25\%}-\varepsilon_{fM0.05\%}}$$

where $\sigma_{fM0.25\%}$ and $\varepsilon_{fM0.25\%}$ are the measured tensile strength and strain at the strain value $\varepsilon_{fM}=0.25\%$, $\sigma_{fM0.05\%}$ and $\varepsilon_{fM0.05\%}$ are the measured tensile strength and strain at the strain value $\varepsilon_{fM}=0.05\%$.

The fracture toughness values of the composites were determined following ISO 13586:2000 standard using single-edge-notched bend (SENB) specimens (60.0 mm long × 8.0 mm wide × 3.0 mm thick). A sharp notch was machined at the midpoint of each specimen (4 mm deep). A natural pre-crack was generated by tapping a new razor blade into the notch. SENB specimens were also tested by Instron 3367 using a three-point-bending rig. Owing to the brittle nature of epoxy, the test speed was set to 0.5 mm min^−1^ to achieve sufficient loading time before the end of each test. The critical stress intensity factor ($K_{IC}$) was calculated by using Equation (7) [45] as follows:

(7) $$K_{IC}=f\left(a/w\right)\frac{F_{Q}}{h\sqrt{w}}$$

where $F_{Q}$ is the maximum load of the load–displacement curve for SENB specimens, h is the thickness of the specimen, w is width, and a denotes sharp crack of length between 0.45w and 0.55w. The f(a/w) is related to the geometry of the sample and can be calculated by using Equations (8) and (9) [45] as follows:

(8) $$f\left(a/w\right)=6\alpha^{1/2}\frac{1.99-\alpha \left(1-\alpha \right)\left(2.15-3.93\alpha +2.7\alpha^{2}\right)}{\left(1+2\alpha \right){\left(1-\alpha \right)}^{3/2}}$$

(9) α=a/w

The critical energy release rate (*G_IC_*) is calculated as Equation (10) [46]:

(10) $$G_{IC}=K_{IC}^{2}\left(\frac{1-\nu^{2}}{E}\right)$$

where *E* is the elastic modulus of the MXene/epoxy composites, *ν* denotes Poisson’s ratio of DGEBA/MTHPA system. The value of 0.29 was used [47].

Micromorphology of specimens was gold-coated and observed at 5 kV by scanning electron microscope (SEM, JEOL JSM-7600 F, Tokyo, JPN). Microstructures of Ti_3_C_2_T_x_ were examined by transmission electron microscopy (TEM, JEOL JEM-2100 F, Tokyo, JPN). For composites, the samples were cut using an ultramicrotome (LKB Nova, Bromma, SWE) equipped with a diamond knife. Thin sections with a thickness of ~100 nm were then cut from mesa of about 1 × 1 mm^2^ and collected on 200 mesh copper grids.

## 3. Results and Discussion

**Figure 2:**
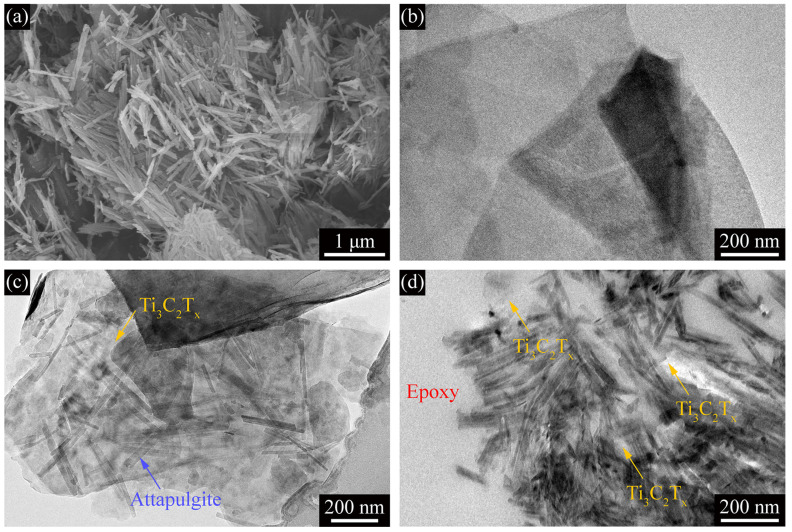
SEM image for (**a**) ATP, and TEM images for (**b**) Ti_3_C_2_T_x_, ATP–MXene hybrids in (**c**) M02A025/epoxy and (**d**) M02A10/epoxy.

SEM and TEM images in Figure 2 show the morphologies of ATP, Ti_3_C_2_T_x_ and their hybrids with different loads of ATP. As shown in Figure 2a, ATP particles were needle-like 1D rigid rods. The dimensions for ATP nanorods were mainly 0.2–1 μm long and roughly 10–20 nm in diameter. As depicted in Figure 2b, MXene layers were 2D platelet-like and stacked by few layers. As shown in Figure 2c,d, comparing the morphology of the reinforcement in the composite slices, it can be clearly seen that the direction of ATP was randomly adsorbed onto the surface of MXene. The exposed area of the MXene sheet was inversely proportional to the amount of ATP added. ATP–MXene hybrids were observed in the cured epoxy composites, which indicated that ATP rods were firmly attached to the surface of MXene lamellae by intermolecular forces (hydrogen bonds), as evidenced by the adsorption that occurred at the edges of MXene lamellae.

**Figure 3:**
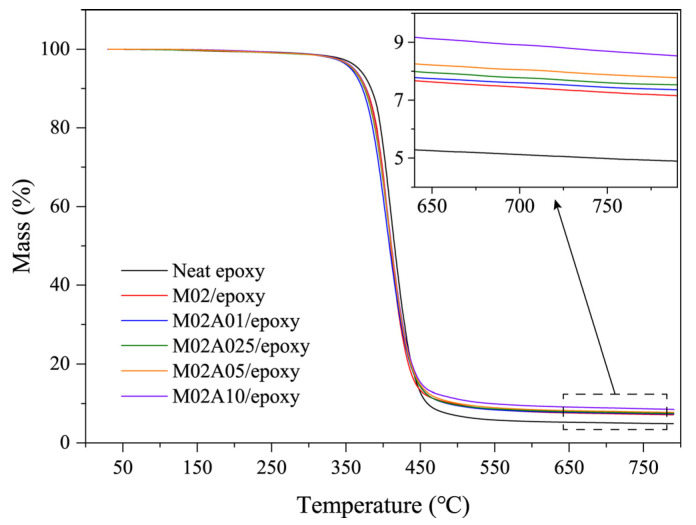
TGA plots of neat epoxy and ATP–MXene hybrids/epoxy composites under a nitrogen atmosphere.

TGA was utilized to investigate the thermal stability of each nanocomposite under a nitrogen atmosphere (Figure 3). All TGA curves exhibit one main degradation stage. The sharp mass loss in the range of 350–450 °C was mainly due to the decomposition of the epoxy matrix. Compared to neat epoxy, the composite of each component decreased slightly when near 400 °C, which should be caused by the destruction of the hydrogen bond between the ATP–MXene hybrids and the epoxy at high temperatures. All samples were proportional to ATP loading at 790 °C, and the differences in residual masses were more significant than that in the masses of added hybrids. The char produced by the decomposition of epoxy could not be completely decomposed at a temperature of 800 °C, which led to adhesion and retention of more char on the filler [48,49]. The results of heating scans are summarized in Table 2.

**Figure 4:**
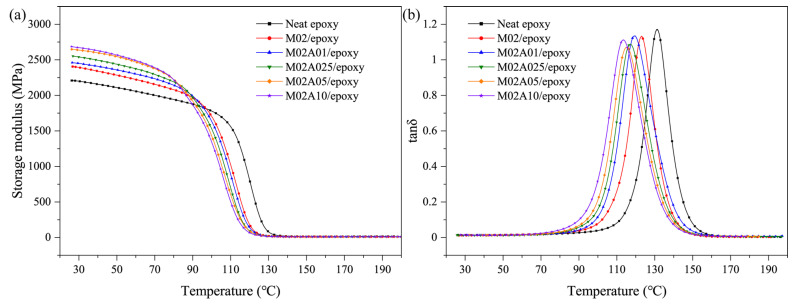
Dynamic mechanical properties of neat epoxy and ATP–MXene hybrids/epoxy composites: (**a**) storage modulus and (**b**) loss angle tangent.

Storage modulus is an index reflecting the elastic properties and influenced by the interfacial interactions between the filler and resin matrix of polymer composites [50]. As shown in Figure 4a, incorporating ATP led to increased storage modulus in the low-temperature range (25–70 °C). The addition of rigid fillers increased the storage modulus. However, the improvement effect of M02A10/epoxy was limited. The ATP/MXene interface was weaker than the ATP/epoxy interface or the MXene/epoxy interface. The load cannot be effectively transferred. When too much ATP was added, agglomerations formed on the surface of MXene (Figure 2d), which increased the number of ATP/MXene interfaces.

Figure 4b illustrates the loss angle tangent (tanδ) of ATP–MXene hybrids/epoxy composites, and the temperature at maximum tanδ value reflected the glass transition temperature (T_g_). Compared to the neat epoxy, all ATP–MXene hybrids/epoxy composites showed lower T_g_ values. Polymer composites have a wide relaxation temperature range [51,52,53,54]. The storage modulus value at room temperature, T_g_ and crosslinking density is shown in Table 3. ATP–MXene hybrids restricted the slippage of adjacent epoxy chains through strong bonding, making the connections around the reinforcement closer and reducing the crosslinking density of the epoxy network of the whole composites. The crosslinking density of the epoxy matrix was inversely proportional to the content of the hybrids, resulting in a gradual decrease in the T_g_ value as the amount of the hybrids increased.

**Figure 5:**
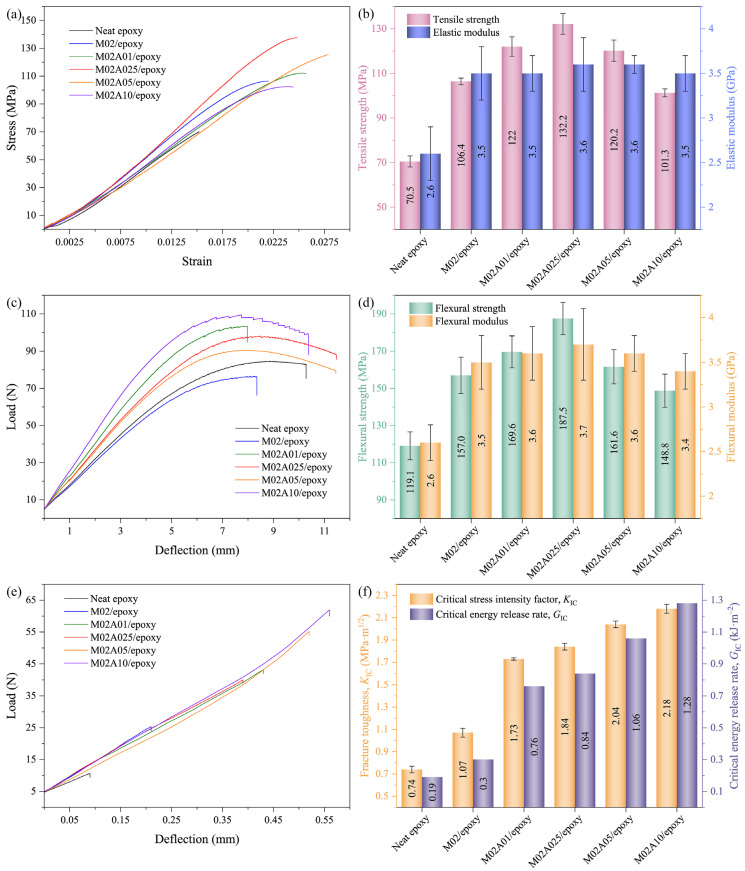
(**a**) Typical stress–strain curves in tension, (**b**) elastic modulus and tensile strength, (**c**) typical load-deflection curves in bending, (**d**) flexural modulus and flexural strength, (**e**) typical load-deflection curves in fracture toughness, (**f**) fracture toughness and critical energy release rate values of fracture toughness of ATP–MXene hybrids/epoxy composites.

Figure 5a,b shows the tensile strength, elastic modulus, flexural strength and flexural modulus of neat epoxy and ATP–MXene hybrids/epoxy composites at different ATP loadings, respectively. The addition of ATP enhanced the mechanical properties of the composites when compared to M02/epoxy. With the increase of the ATP content, an increasing trend of properties was first shown. This was followed by a decrease. At 0.25 wt % ATP content, all mechanical properties of the ATP–MXene hybrids/epoxy composites reached maximum values. Compared to M02/epoxy, tensile strength (106.4 MPa) and elastic modulus (3.5 GPa) increased by 24% (132.2 MPa) and 3% (3.6 GPa), while the flexural strength (157.0 MPa) and flexural modulus (3.5 GPa) increased by 19% (187.5 MPa) and 6% (3.7 GPa), respectively. Compared to neat epoxy, the mechanical properties significantly improved, tensile strength and elastic modulus increased by 88% and 38%. In comparison, the flexural strength and flexural modulus increased by 57% and 42%, respectively. The mechanical properties for neat epoxy and its composites with different filler loadings are summarized in Table 4. Considering the enhancement effects of MXene [15,37,55], graphene [56] and graphene oxide (GO) [46,57,58,59,60], the enhancement of ATP–MXene hybrids was also comparable, as shown in Table 5.

**Figure 6:**
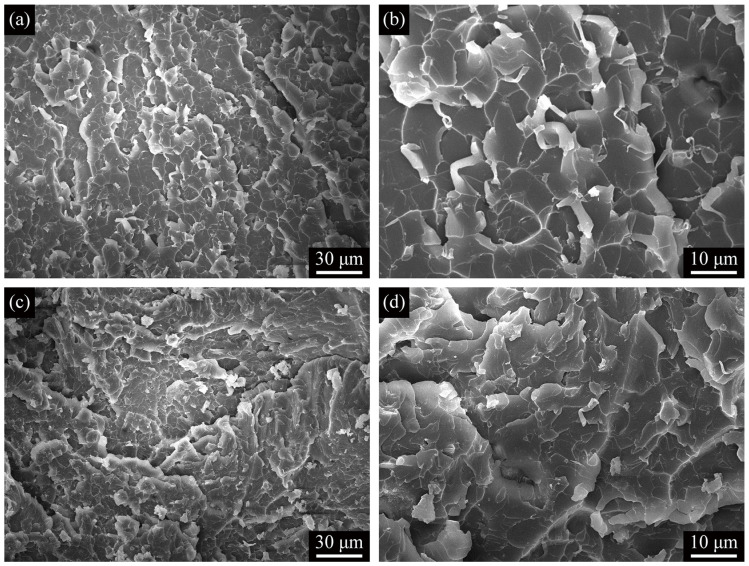
(**a**) Low-magnification and (**b**) high-magnification SEM images of tensile fracture surfaces of M02A025/epoxy; (**c**) Low-magnification and (**d**) high-magnification SEM images of tensile fracture surfaces of M02A10/epoxy.

SEM was used to study the fracture behaviors of the ATP–MXene hybrids/epoxy composites after tensile tests (Figure 6). The tensile fracture surfaces of ATP–MXene hybrids/epoxy composites showed multiplane features with many tortuous cracks, which indicates that the incorporated ATP–MXene hybrids had induced the deflection of propagating crack fronts. The crack deflection process can arouse off-plane loading and generate new fracture surfaces, increasing the required strain energy for crack propagation. Compared with M02A025/epoxy (Figure 6a,b), the number of scaly regions in the fracture surface of M02A10/epoxy (Figure 6c,d) was more. Still, the area was reduced, which also indicated that the increase of ATP led to the change of crosslink density. In addition, due to the agglomeration of the reinforcement, many separations between the substrates could be observed in M02A10/epoxy (Figure 6c), which was the main reason for the decrease in tensile properties of M02A10/epoxy.

Fracture toughness of ATP–MXene hybrids/epoxy composites at different filler loadings were evaluated by the SENB method, as shown in Figure 5c. The fracture toughness value was positively correlated with the amount of added ATP. M02A10/epoxy had the largest fracture toughness value (2.18 MPa·m^1/2^), which was 104% higher than M02/epoxy (1.07 MPa·m^1/2^). It was significantly 195% higher than neat epoxy (0.74 MPa·m^1/2^). The propagation of crack was severely hindered through the ATP–MXene hybrids, which led to the dissipation of higher fracture energy in composites. Further, the critical energy release rate (*G_IC_*) of M02A10/epoxy had increased dramatically by ~570% than neat epoxy.

**Figure 7:**
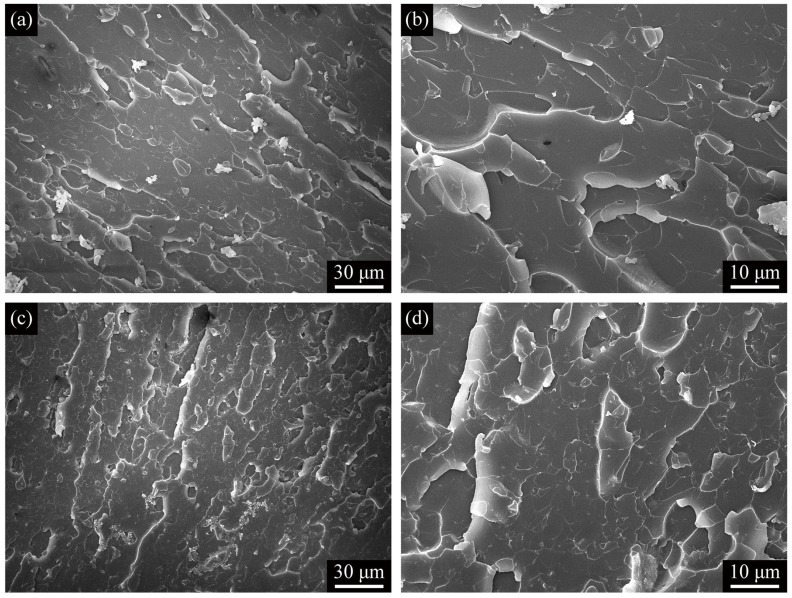
(**a**) Low-magnification and (**b**) high-magnification SEM images of SENB flexural fracture surfaces of M02A025/epoxy; (**c**) Low-magnification and (**d**) high-magnification SEM images of SENB flexural fracture surfaces of M02A10/epoxy.

Figure 7 shows SENB flexural fracture surfaces of the M02A025/epoxy and M02A10/epoxy composites. Compared to M02A025/epoxy (see Figure 7a,b), it can be seen that the addition of ATP to the epoxy increased the toughness of the composite, making the fracture surface much rougher (see Figure 7c,d). The main reason was that due to excessive ATP, the ATP, which could not be completely adsorbed on the MXene layer, was also dispersed in the matrix. Regardless of the tensile fracture in Figure 6 or the SENB flexural fracture in Figure 7, few debonded reinforcing phases were observed. The observation of this failure mode indirectly reflected the solid interfacial interaction between the reinforcement and the epoxy matrix.

As shown in Table 2, although the flexural modulus of elasticity of neat epoxy resin was equal to the tensile modulus of elasticity, the flexural strength was much higher than the tensile strength. Different from the three-point stress state of the bending test, any defects in the parallel section of the tensile specimen may have caused the failure of the specimen, so that any part of the parallel section of the test specimen may have had a fracture surface; However, the failure of the bending specimen under the action of the upper indenter often occurred in the center of the specimen, which was less affected by the defects than the tensile test. This was the reason why the flexural strength was greater than the tensile strength. Although the flexural modulus and elastic modulus of the composites were slightly different under the action of the reinforcing phase, the changing trend and regular pattern of the two were the same. In general, similar results could be seen in the relevant reports of epoxy matrix composites [37,56,57,61]. By comparing the results of tensile strength, flexural strength and fracture toughness, it was found that the same hybrids content improves the two properties inconsistently, which was also caused by different test methods. The results of the tensile and flexural test depended on the properties of the relatively large sample. Regardless of the nature of the sample, a single defect in the sample (such as a large agglomerate) could cause failure. In contrast, the sample damaged after the SEBN fracture test was carried out along the pre-crack, so it was not easily affected by a single defect. Therefore, when the content of ATP exceeded 0.25 wt %, the tensile and flexural strength decreased due to aggregation and other reasons, but it could continue to strengthen the fracture toughness of the material. It needs to be emphasized that the uniform dispersion of fillers was critical to the system performance because a single large defect/agglomerate could eliminate the toughening of the surrounding matrix. The inconsistency in flexure strength and fracture toughness improvements was a common phenomenon [56,57,62].

## 4. Conclusions

The ATP–MXene hybrids with 1D ATP nanorods attached on 2D Ti_3_C_2_T_x_ nanoplatelets at various combinations were prepared through a solution process, and the epoxy composites with uniformly distributed ATP–MXene hybrid fillers were fabricated. The experimental results showed that ATP can connect onto the MXene surface to form hybrids, which increases the surface roughness of MXene. The addition of ATP led to different nanorods coverage on MXene lamellae, resulting in different interfacial strengths of the hybrids, which affected the mechanical properties of the composites. The greatest improvement in mechanical properties appeared when the hybrids were M02A025 since a stronger interfacial interaction was achieved. These properties were superior to composites incorporated by MXene individually. The formation of ATP–MXene hybrids avoided the need to change the surface functional groups of MXene and further improved the mechanical properties of MXene/epoxy composites. Consequently, the ATP–MXene hybrids showed the application potential for acquiring high-performance mechanical reinforcements.

## Figures and Tables

**Table 1:** Composition of the formulations studied in this work.

Sample Abbreviation	Formulation
Neat epoxy	(DGEBA (16.216 g) and MTHPA (13.784 g) at mass ratio of 100:85) + 0.3 wt % DMP-30 (0.090 g)
M02/epoxy	0.2 wt % Ti_3_C_2_T_x_ (0.060 g) + (DGEBA (16.216 g) and MTHPA (13.784 g) at mass ratio of 100:85) + 0.3 wt % DMP-30 (0.090 g)
M02A01/epoxy	0.2 wt % Ti_3_C_2_T_x_ (0.060 g) + 0.1 wt % ATP (0.030 g) + (DGEBA (16.216 g) and MTHPA (13.784 g) at mass ratio of 100:85) + 0.3 wt % DMP-30 (0.090 g)
M02A025/epoxy	0.2 wt % Ti_3_C_2_T_x_ (0.060 g) + 0.25 wt % ATP (0.075 g) +(DGEBA (16.216 g) and MTHPA (13.784 g) at mass ratio of 100:85) + 0.3 wt % DMP-30 (0.090 g)
M02A05/epoxy	0.2 wt % Ti_3_C_2_T_x_ (0.060 g) + 0.5 wt % ATP (0.151 g) + (DGEBA (16.216 g) and MTHPA (13.784 g) at mass ratio of 100:85) + 0.3 wt % DMP-30 (0.090 g)
M02A10/epoxy	0.2 wt % Ti_3_C_2_T_x_ (0.060 g) + 1.0 wt %. ATP (0.304 g) + (DGEBA (16.216 g) and MTHPA (13.784 g) at mass ratio of 100:85) + 0.3 wt % DMP-30 (0.090 g)

**Table 2:** Thermal degradation parameters determined by TGA of the neat epoxy and ATP–MXene hybrids/epoxy composites.

Sample	Char Yield (%)	Onset Temperature (°C)	Step Inflection Point (°C)	End Temperature (°C)
Neat epoxy	4.89	382.67	411.55	445.84
M02/epoxy	7.15	378.70	405.96	437.73
M02A01/epoxy	7.36	371.30	403.61	440.25
M02A025/epoxy	7.53	373.65	407.04	441.70
M02A05/epoxy	7.77	372.74	406.50	442.78
M02A10/epoxy	8.53	378.70	409.57	439.71

**Table 3:** Dynamic mechanical properties and crosslinking density of neat epoxy and ATP–MXene hybrids/epoxy composites.

Samples	Storage Modulus (MPa) (RT)	T_g_ (°C)	Storage Modulus (MPa) (T_g_ + 40 °C)	Crosslinking Density (mol/cm^3^)
Neat epoxy	2209	131.3	15	1.35 × 10^−3^
M02/epoxy	2405	122.9	12	1.10 × 10^−3^
M02A01/epoxy	2460	119.3	10	0.93 × 10^−3^
M02A025/epoxy	2552	117.1	11	1.03 × 10^−3^
M02A05/epoxy	2649	115.1	10	0.94 × 10^−3^
M02A10/epoxy	2686	113.2	9	0.85 × 10^−3^

**Table 4:** Mechanical and thermal properties of neat epoxy and its composites.

Samples	Tensile Strength (MPa)	Elastic Modulus (GPa)	Flexural Strength (MPa)	Flexural Modulus (GPa)	*K_IC_* (MPa·m^1/2^)
Neat epoxy	70.5 ± 6.5	2.6 ± 0.3	119.1 ± 7.5	2.6 ± 0.2	0.74 ± 0.03
M02/epoxy	106.4 ± 1.5	3.5 ± 0.3	157.0 ± 8.7	2.6 ± 0.2	1.07 ± 0.04
M02A01/epoxy	122 ± 4.4	3.5 ± 0.2	169.6 ± 9.1	3.5 ± 0.3	1.73 ± 0.01
M02A025/epoxy	132.2 ± 4.6	3.6 ± 0.3	187.5 ± 8.9	3.6 ± 0.2	1.84 ± 0.03
M02A05/epoxy	120.2 ± 4.8	3.6 ± 0.1	161.6 ± 8.5	3.7 ± 0.3	2.04 ± 0.03
M02A10/epoxy	101.3 ± 1.8	3.5 ± 0.2	148.8 ± 8.9	3.6 ± 0.2	2.18 ± 0.04

**Table 5:** Comparison of the tensile strength (*σ*), flexural strength (*σ_fM_*) and fracture toughness (*K_IC_*) of various composite systems and the relative increments after incorporation of MXene, graphene and graphene oxide.

Reinforcement Filler Content (wt %)	*σ*, Gain in *σ* (%), (Matrix *σ* (MPa))	*σ_fM_*, Gain in *σ_fM_* (%), (Matrix *σ_fM_* (MPa))	*K_IC_*, Gain in *K_IC_* (%), (Matrix *K_IC_* (MPa·m^1/2^))	Reinforcement Filler and References
0.2 MXene + 0.25 ATP	132.2, 88, (70.5)	187.5, 57, (119.1)	1.84, 149, (0.74)	ATP–MXene hybrids, this work
0.2 MXene + 1.0 ATP	101.3, 44, (70.5)	148.8, 25, (119.1)	2.18, 195, (0.74)	
0.2	106.4, 51, (70.5)	157, 32, (119.1)	1.07, 45, (0.74)	Ti_3_C_2_T_x_ [37]
1	76.1, 8, (70.5)	128.6, 8, (119.1)	1.41, 91, (0.74)	
1	-	98, 66, (59)	-	Ti_2_CT_x_ [15]
1.2	~66, 24.9, (~53)	-	-	Ti_3_C_2_T_x_ [55]
0.1	~51, −2, (~52)	~152, 8.6, (~140)	0.611, 24, (0.493)	Graphene, thermally reduced GO [56]
0.1	~71, 9, (~65)	~150, 20 (~125)	~96, 28 (~0.75)	Pristine GO sheet [57]
0.5	~75, 15, (~65)	~156, 25, (~125)	~1.22, 63 (~0.75)	
0.1	~75, 6, (~70)	-	~1.9, 27, (~1.5)	Pristine GO sheet [58]
0.5	~70.7, 1, (~70)	-	~−1.2, 20, (~1.5)	
0.1	78.9, 13.0, (69.7)	-	1.14, 12, (1.02)	3-aminopropyl trimethoxysilane functionalized GO sheet [46]
0.2	81.2, 16.5, (69.7)	-	1.22, 20, (1.02)	
0.1	77.9, 11.7, (69.7)	-	1.37, 34, (1.02)	3-glycidoxypropyl trimethoxysilane functionalized GO sheet [46]
0.2	79.2, 13.6, (69.7)	-	1.46, 43, (1.02)	
0.3	66.38, 22, (54.39)	414.10, 87, (221.96)	-	Polyurethane, pristine GO sheet [59]
0.1	94.79, 79, (52.98)	-	0.684, 36, (0.503)	Functionalized GO sheet [60]
0.5	85.51, 61, (52.98)	-	0.669, 33, (0.503)	

## Data Availability

The data presented in this study are available on request from the corresponding author.
